# Sustained Hyperglycemia and Its Relationship with the Outcome of Hospitalized Patients with Severe COVID-19: Potential Role of *ACE2* Upregulation

**DOI:** 10.3390/jpm12050805

**Published:** 2022-05-17

**Authors:** Jose R. Vargas-Rodriguez, José J. Valdés Aguayo, Idalia Garza-Veloz, Jacqueline Martinez-Rendon, Maria del Refugio Rocha Pizaña, Griselda A. Cabral-Pacheco, Vladimir Juárez-Alcalá, Margarita L. Martinez-Fierro

**Affiliations:** 1 Molecular Medicine Laboratory, Unidad Academica de Medicina Humana y C.S, Campus UAZ siglo XXI-L1, Universidad Autónoma de Zacatecas, Zacatecas 98160, Mexico; jrvr159@gmail.com (J.R.V.-R.); josejuan104@gmail.com (J.J.V.A.); idaliagv@uaz.edu.mx (I.G.-V.); jamare85@gmail.com (J.M.-R.); gris91.edia@gmail.com (G.A.C.-P.); vladimir.j.a@uaz.edu.mx (V.J.-A.); 2 Escuela de Ingenieria y Ciencias, Tecnologico de Monterrey Campus Puebla, Puebla 72453, Mexico; mrochap@tec.mx

**Keywords:** COVID-19, SARS-CoV-2, hyperglycemia, diabetes mellitus, *ACE2*

## Abstract

Chronic hyperglycemia increases the risk of developing severe COVID-19 symptoms, but the related mechanisms are unclear. A mean glucose level upon hospital admission >166 mg/dl correlates positively with acute respiratory distress syndrome in patients with hyperglycemia. The objective of this study was to evaluate the relationship between sustained hyperglycemia and the outcome of hospitalized patients with severe COVID-19. We also evaluated the effect of high glucose concentrations on the expression of angiotensin-converting enzyme 2 (*ACE2*). We carried out a case-control study with hospitalized patients with severe COVID-19 with and without sustained hyperglycemia. In a second stage, we performed in vitro assays evaluating the effects of high glucose concentrations on *ACE2* gene expression. Fifty hospitalized patients with severe COVID-19 were included, of which 28 (56%) died and 22 (44%) recovered. Patients who died due to COVID-19 and COVID-19 survivors had a high prevalence of hyperglycemia (96.4% versus 90.9%), with elevated central glucose upon admission (197.7 mg/dl versus 155.9 mg/dl, *p* = 0.089) and at discharge (185.2 mg/dl versus 134 mg/dl, *p* = 0.038). The mean hypoxemia level upon hospital admission was 81% in patients who died due to COVID-19 complications and 88% in patients who survived (*p* = 0.026); at the time of discharge, hypoxemia levels were also different between the groups (68% versus 92%, *p* ≤ 0.001). In vitro assays showed that the viability of A549 cells decreased (76.41%) as the glucose concentration increased, and the *ACE2* gene was overexpressed 9.91-fold after 72 h (*p* ≤ 0.001). The relationship between hyperglycemia and COVID-19 in hospitalized patients with COVID-19 plays an important role in COVID-19-related complications and the outcome for these patients. In patients with chronic and/or sustained hyperglycemia, the upregulation of *ACE2*, and its potential glycation and malfunction, could be related to complications observed in patients with COVID-19.

## 1. Introduction

In December 2019, cases of pneumonia of unknown etiology began to emerge in the city of Wuhan, Hubei province, China. Subsequent in-depth analysis of samples obtained from the lower respiratory tract revealed a novel coronavirus as the causative agent, called severe acute respiratory syndrome coronavirus 2 (SARS-CoV-2), which causes coronavirus disease 2019 (COVID-19) [1]. By April 2022, more than 500 million confirmed cases and more than 6 million deaths have been recorded globally [2]. The pathophysiological mechanism of this disease is not yet completely defined [1], but comorbidities such as diabetes mellitus (DM), chronic lung or kidney disease, systemic arterial hypertension (SAH) [1,3,4,5] and obesity [3] increase the risk of complications and death in patients with COVID-19 [6].

DM has turned out to be the greatest risk factor for hospital admission, severe pneumonia [7,8], acute respiratory syndrome severity and worse prognosis in patients with COVID-19 [9,10]. In addition to SARS-CoV-2 infection [7,11], DM also constitutes a poor prognostic factor for patients with other viral infections such as SARS-CoV [12], Middle Eastern respiratory syndrome-related coronavirus (MERS-CoV) [8,13] and influenza A/H1N1 [14,15]. Patients with uncontrolled DM (glycated hemoglobin [HbA1c] > 9%) have a 2.3-fold increased risk of experiencing the severe forms of COVID-19, a 2.5-fold increased risk of death associated with COVID-19 [7,11] and a 60% increased risk of over-aggregated nosocomial pneumonia [15]. Thus, the presence of hyperglycemia in these patients upon hospital admission should not be overlooked and should be treated appropriately to improve the prognosis of patients with COVID-19 with or without DM [16].

Stress-induced hyperglycemia may be defined as any elevation of serum glucose upon hospital admission in patients with or without previously diagnosed DM [17] or as two abnormal glucose measurements ≥126 mg/dl or HbA1c ≥ 6.5% or glucose ≥ 200 mg/dl with symptoms of hyperglycemia in the absence of a previous history of DM [6,13,18]. In patients with hyperglycemia, more severe forms of infections with SARS-CoV, MERS-CoV [19,20] or SARS-CoV-2 have been observed [16,20,21]. These findings may be related in part to the immunosuppressive effect of hyperglycemia, with modifications of the innate and humoral immune response [7] via reduced function of macrophages, lymphocytes and neutrophils [20]. These altered functions are combined with the inflammatory processes of the infection itself, increasing the risk of COVID-19 complications [19]. The more aggressive infection by SARS-CoV-2 observed in patients with hyperglycemia without a previous diagnosis of DM may be related to poor or even absent glycemic control upon hospital admission [6,22].

The entry of SARS-CoV-2 into the host is mediated by the binding of the viral S glycoprotein to its membrane receptor [23]. SARS-CoV-2 interacts directly with angiotensin-converting enzyme 2 (*ACE2*) through the B domain of the S glycoprotein [23,24]. Within the lung, the highest expression of *ACE2* occurs in type II pneumocytes [22,23]. Internalization of *ACE2* promoted by SARS-CoV-2 results in a loss of this enzyme on the membrane surface, avoiding the degradation of angiotensin II (Ang II) to angiotensin 1–7 (Ang 1–7) [7,25]. Another mechanism related to decreased *ACE2* expression on the membrane surface is sustained uncontrolled hyperglycemia observed in patients with DM, probably induced by enzyme glycosylation [26], which could also regulate *ACE2*–S binding [27,28]. This action would modulate the attachment of the virus to the receptor and decrease immune system activity against SARS-CoV-2 [29], contributing to lung injury and fibrosis associated with COVID-19 [7,25] and other respiratory diseases [26].

There have been reports of increased *ACE2* expression in the lungs of diabetic mice, but surprisingly *ACE2* expression levels returned to normal after the administration of insulin [29,30]. Similarly, upregulation and non-enzymatic glycation of *ACE2* in DM cardiomyocytes could increase the susceptibility of patients with DM to COVID-19 by favoring the cellular entry of SARS-CoV-2 [28]. These findings suggest the critical role of *ACE2* in the pathophysiology of SARS-CoV-2 infection. Hence, the objective of this study was to determine the relationship between sustained hyperglycemia and the outcome of hospitalized patients with severe COVID-19 and the influence of high glucose concentrations on *ACE2* gene expression.

## 2. Materials and Methods

### 2.1. Study Design and Study Population

This was a case-control study in which data from patients were obtained retrospectively. The study included patients over 18 years of age positive for SARS-CoV-2 based on real-time reverse transcriptase-polymerase chain reaction (qRT-PCR) assay, with hospitalization criteria and with a diagnosis of severe COVID-19. The study was carried out in the Emergency Medical Specialties Unit belonging to the health services of Zacatecas, Mexico, from March to August 2021. The study included patients with a central glucose registry upon admission and discharge, plus the reason for discharge (improvement or death). The exclusion criteria for the study were patients with an incomplete medical record, patients with diagnoses of other primary infections or with concomitant diseases related to the previous administration of immunosuppressive drugs. A total of 50 patients were included, of which 28 were discharged due to death from COVID-19 complications and 22 were discharged due to clinical improvement.

Study approval was obtained from the research committee of the Academic Unit of Human Medicine and Health Sciences of the Autonomous University of Zacatecas Francisco García Salinas and by the research and ethics committee of the General Hospital of Zacatecas Luz González Cosío (ID number: 0223/2021/C), in Zacatecas, Mexico.

### 2.2. Clinical Information

For this study, general data including the sex and age of each patient and the date and reason for hospital admission and discharge, and clinical data such as a personal history of DM, hypertension, obesity and respiratory diseases not related to SARS-CoV-2 were taken into account. Glucose levels and oxygen saturation upon admission and discharge, the need for supplemental oxygen therapy and/or the need for orotracheal intubation, laboratory results and medical treatment received were also collected from the clinical record of each patient.

### 2.3. Cell Culture and Cell Viability Testing

In vitro cell culture assays were performed by using A549 cells—human epithelial lung cells (pneumocytes)—cultured in a 75 cm^2^ sterile culture flask (Corning, Corning, NY, USA, product number 430720U) with F-12K culture medium (ATCC, product number 30-2004) supplemented with fetal bovine serum (10%) (Byproducts, 17001) and antibiotic-antimycotic containing 10,000 U/mL penicillin, 10,000 mg/mL streptomycin, and 25 mg/mL am-phot ricin B (1%) (Gibco, Waltham, MA, USA, product number 15240062). Upon reaching 90–100% confluence, cells were sub-cultured into 96-well plates (Corning, product number 3797), with 1.5 × 10^4^ cells per well. Cells were incubated with a complete medium adjusting the glucose concentrations to 50, 100, 200, 400, 800 or 1000 mg/dl for 24, 48 or 72 h, renewing the medium daily. After treatment with glucose at different concentrations and different exposure times to maintain stable glucose concentration, cell viability was examined by using the Alamar Blue reagent (Invitrogen, Waltham, MA, USA, product number DAL1100) with incubation for 2 h and a color change measured at 590 nm in a Smartreader 96 spectrophotometer (Accuris Instruments, Edison, NJ, USA). The cytotoxicity after glucose treatment was measured by using the following formula [31]:

*Cytotoxicity* (%) = (*A experimental group*) − (*A low control*) × 100 (*A high control*) − (*A low control*)

where *A* is absorbance, *experimental group* is the cell culture plus Alamar Blue, *high control* is the cell culture without treatment plus Alamar Blue and *low control* is culture medium without cells plus Alamar Blue.

### 2.4. RNA Extraction and Complementary DNA (cDNA) Synthesis from A549 Cells

After quantifying cell viability, RNA was extracted from A549 cells with TRI Reagent^®^ LS (TS 120) according to the manufacturer’s indications for adherent cells. The extraction quantity and quality were verified with electrophoresis of the samples in a 1% agarose gel and by using a Nanodrop 1000 spectrophotometer (Thermo Fisher Scientific, Waltham, MA, USA). Reverse transcription was performed using 1000 ng of RNA with the High-Capacity cDNA Reverse Transcription Kit (Applied Biosystems, Waltham, MA, USA, product number 4368814) according to the protocol established by the manufacturer. The thermal cycling conditions were 25 °C for 10 min, 37 °C for 120 min and 85 °C for 5 min. cDNA was stored at −20 °C until use.

### 2.5. Measurement of ACE2 Messenger RNA (mRNA) Expression by qRT-PCR

*ACE2* mRNA expression was determined by using a StepOne Plus Real-Time PCR System (Applied Biosystems). The 10-μL reactions contained 1X SYBR Green reagent (Thermo Scientific) with the forward primer (5′-CATTGGAGCAAGTGTTGGATCTT-3′) and the reverse primer (5′-GAGCTAATGCATGCCATTCTCA-3′), which generates a 108-bp amplicon of the *ACE2* gene. The ribosomal protein lateral stalk subunit P0 (*RPLP0*) gene was used as a reference gene (forward primer: 5′-TGGTCATCCAGCAGGTGTTCGA-3′; reverse primer: 5′-ACAGACACTGGCAACATTGCGG-3′), which amplified a 119-bp fragment. The thermal cycling conditions for qRT-PCR were 95 °C for 15 min, followed by 40 cycles at 95 °C for 15 s, 60 °C for 30 s and 72 °C for 30 s. Standard curves both for *RLP0* and *ACE2* and melting curve analysis were performed at the end of each run to confirm the efficiency and specificity of the products (supplementary Figure S1A–D).

All samples were run in duplicate, including two negative controls for each gene. *ACE2* mRNA expression was determined by using the 2^−ΔΔCq^ method [32,33].

### 2.6. Statistical Methods

Microsoft^®^ Excel was used to collect and organize data and SigmaPlot v12.0 Software (Systat Software Inc., San Jose, CA, USA) was used for statistical analysis. The statistical tests used for data analysis and to determine differences between study groups were Student’s t-test for comparison between two groups, analysis of variance (ANOVA) for comparison between more than two groups and measures of central tendency and dispersion. A *p* value < 0.05 was considered statistically significant.

## 3. Results

### 3.1. Characteristics of the Study Population

The study population comprised 50 participants diagnosed with severe COVID-19 according to the criteria established by the official Mexican health guidelines: positive diagnosis of SARS-CoV-2 infection by RT-PCR, clinical signs of pneumonia plus tachypnoea and severe shortness of breath or oxygen saturation (SpO_2_) <90% [34]. Table 1 displays the general characteristics of the study population. Of the 50 participants, 29 (58%) were women. Of the women, 19 (65.5%) died due to COVID-19 complications and 10 (34.5%) recovered. Twenty-one (42%) participants in the study were men, of whom 9 (42.9%) died due to COVID-19 complications and 12 (57.1%) were discharged because of improvement. There were no sex differences in the number of patients who died or survived (*p* = 0.616).

Considering the reason for discharge, 44% of the patients recovered after their hospitalization and 56% of the patients died (Table 1). The mean age for the recovered patients was 55.9 years (range 36–80 years) and the mean age of patients who died was 59.4 years (range 38–86 years) (*p* < 0.001). The hospital stay was longer in patients who died (21 ± 18 days) compared with those who recovered (10 ± 8 days) (*p* = 0.004). There were no significant differences in COVID-19-related symptoms between the patients who died or recovered.

### 3.2. Clinical and Biochemical Parameters

DM, arterial hypertension and obesity were commonly observed in patients hospitalized for COVID-19 (Table 2).

Stress hyperglycemia was also a frequent finding in these patients, with a frequency of 68.2% in recovered patients and 60.7% in patients who died (*p* = 0.803). The need for mechanical ventilation was a risk factor for death in the studied population (odds ratio [OR] = 36.7, 95% confidence interval [CI]: 6.6–202.9, *p* < 0.001). Hyperglycemia (>160 mg/dl glucose) upon discharge was also associated with death (OR = 5.3, 95% CI: 1.5–18.4, *p* = 0.016).

**Figure 1:**
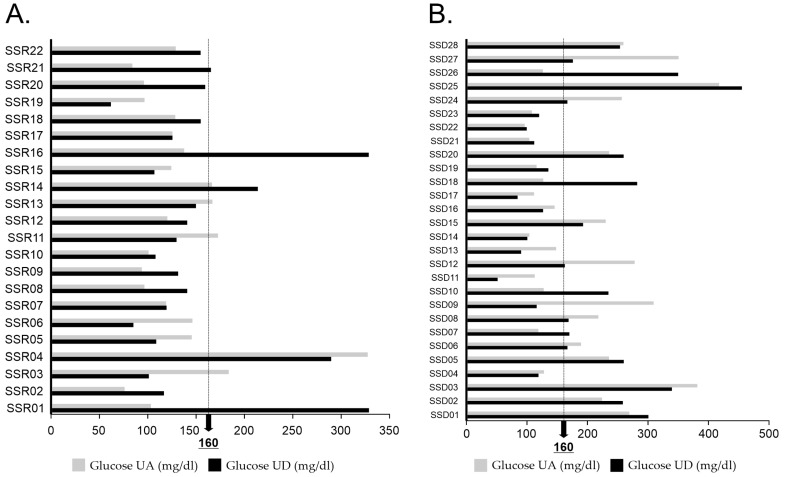
Glucose levels in the study population classified as patients who recovered (**A**) and patients who died due to COVID-19 complications (**B**). Both groups had a high prevalence of hyperglycemia upon admission; most patients who died (60.7%) had sustained hyperglycemia ≥160 mg/dl even with insulin treatment. The majority of recovered patients (77.3%) had glucose levels <160 mg/dl upon discharge. SSD, study subject deceased; SSR, study subject recovered; UA, upon admission; UD, upon discharge.

The mean central glucose of patients who died was 197.7 ± 92.6 mg/dl upon admission, while for patients who recovered it was 155.9 ± 72.9 mg/dl (*p* = 0.197). All patients with hyperglycemia were treated with intravenous insulin according to the Standards of Medical Care in Diabetes 2021 guidelines [35]. Hyperglycemia persisted until discharge, with a mean glucose level of 191.0 ± 95.9 mg/dl for patients who died and 134.0 ± 52.9 mg/dl for patients who recovered (*p* = 0.038) (Figure 1).

Oxygen saturation was lower in patients who died due to COVID-19 complications (81% ± 11%) compared with patients who recovered (88% ± 9%) (*p* = 0.019), with a worse progression of hypoxemia in the patients who died (67% ± 17%) and a slight recovery of oxygen saturation in the recovered patients (92% ± 3%) (*p* ≤ 0.001). Regarding oxygen therapy and the need for orotracheal intubation: 46 patients (92%) required supplemental oxygen at some point during their hospitalization. Twenty-two (78.6%) of the patients who died required orotracheal intubation, while only 5 (22.7%) of the patients who recovered required it (*p* < 0.001).

There were cases of over-aggregated pneumonia, with a higher frequency in patients who died due to COVID-19 complications (6 [12%] individuals) than that observed in recovered patients (1 [2%] case) (*p* < 0.001). Of the patients who died with over-aggregated nosocomial pneumonia, 2 patients were admitted with uncontrolled DM, and 4 were admitted with hyperglycemia due to stress and maintained glucose levels ≥160 mg/dl during their hospital stay. The recovered patient was admitted with hyperglycemia due to stress, but upon discharge showed central glucose ≤100 mg/dl.

Arterial hypertension was more frequent in patients who died due to COVID-19 complications (*p* = 0.021), with a 4.8-fold greater risk of death (95% CI: 1.4–16.2, *p* = 0.021). Patients with mechanical ventilation had a 36.7 times greater risk of death and patients with glucose >160 mg/dl had a 5.3 times greater risk of death compared with patients whose glucose levels were <160 mg/dl upon discharge.

### 3.3. High Glucose Treatment Reduced the Viability of A549 Cells

**Figure 2:**
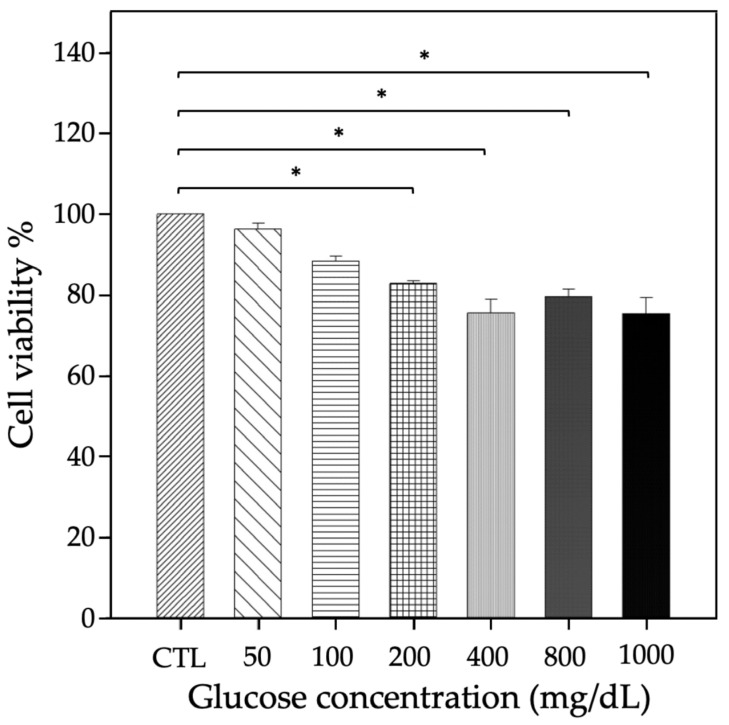
Effect of glucose on the viability of A459 cells (pneumocytes). Cell viability was determined by using the Alamar Blue reagent in cells treated with glucose for 72 h. The different colors in bars represent the control and the concentrations of 50, 100, 200, 400, 800, and 1000 mg/dl of glucose present in the culture medium. There was a decrease in cell viability compared with control cells beginning at 200 mg/dl glucose. The asterisks indicate a statistically significant difference compared to the control group (Dunn’s method); * *p* ≤ 0.05.

Patients with hyperglycemia and/or DM present a worse prognosis after SARS-CoV-2 infection compared with patients without these conditions. To explore the effect of high glucose concentrations on pneumocytes, we performed in vitro cell viability assays using A549 cells (pneumocytes), a model of human lung epithelial cells. These cells were cultured in the presence of different glucose concentrations (50–1000 mg/dl) for 24, 48 or 72 h, and viability was assessed with the Alamar Blue reagent. Beginning at 200 mg/dl, glucose significantly decreased cell viability after 72 h of treatment (Figure 2).

### 3.4. ACE2 mRNA Expression

Previous studies have reported that patients with DM have higher expression of *ACE2* in peripheral blood mononuclear cells [36] and cardiomyocytes [28], and upregulation of *ACE2* expression could increase susceptibility to COVID-19. Hence, *ACE2* gene expression was assessed by qRT-PCR in A549 cells cultured with different glucose concentrations (Table 3). Compared with control of each time, 200, 400 or 800 mg/dl glucose significantly increased *ACE2* expression, with maximum expression in cells treated with 800 mg/dl glucose for 72 h. The *p* values in Table 3 show the effect of different glucose concentrations through study time.

**Figure 3:**
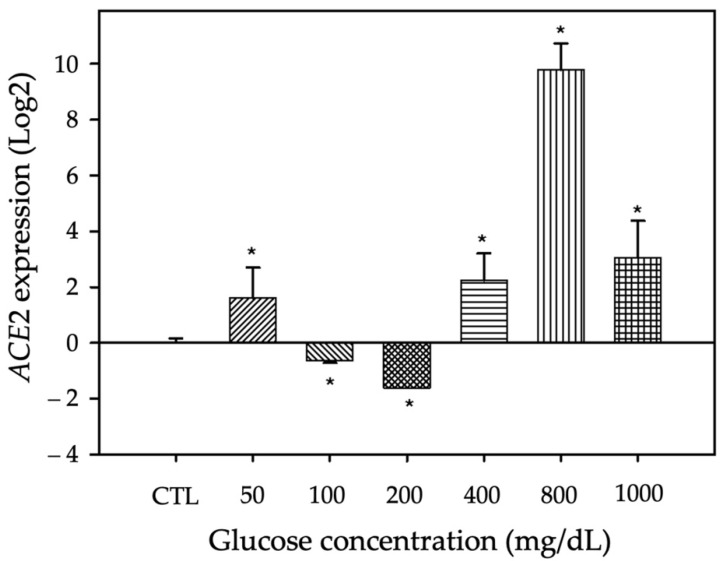
Fold change of *ACE2* expression in A549 cells treated with 50, 100, 200, 400, 800 or 1000 mg/dl glucose for 72 h. Compared with the control, there were significant differences in the *ACE2* expression level in cells treated with 200, 400 or 800 mg/dl glucose. The bars represent the control and the different concentrations of glucose present in the culture medium. Multiple comparison statistical test, followed by multiple comparisons versus control group (Dunnett’s method), indicate a statistically significant difference, * *p* ≤ 0.05.

Figure 3 displays the *ACE2* expression in cultured pneumocytes subjected to sustained glucose treatment for 72 h. This treatment time produced significant fold changes compared with the control group, with maximum expression after treatment with 800 mg/dl glucose (*p* < 0.005).

## 4. Discussion

Acute hyperglycemia is one of the most common acute metabolic complications in patients with DM and is commonly triggered by viral infections (including SARS-CoV-2). Hyperglycemia increases the risk of severity and mortality in these patients [37,38,39,40]. The prevalence of DM in patients with COVID-19 is highly variable due to the multiple factors that influence its presentation and development. Huang et al. [41] reported a DM prevalence of 20% in their study of clinical features in patients with COVID-19 in China, Guan et al. [4] reported 7%, the Chinese Center for Disease Control and Prevention reported 5% [42], Grasseli et al. [43] reported 17% in Italy, Docherty et al. [44] reported 19% in the United Kingdom and Bello-Chavolla et al. [45] reported 18% in Mexico. The prevalence of 30% in our study is higher than the previous report from Mexico.

According to Iacobellis et al. [16], of 85 patients with COVID-19 admitted to the hospital, 27 (32%) reported DM with glucose upon admission of 166 ± 81 mg/dl. The authors found a positive correlation between mean serum glucose and progression of radiographic findings and acute respiratory distress syndrome. We obtained similar results in this study: of the 50 patients hospitalized with severe COVID-19, 15 (30%) reported DM upon admission, of which 10 (20%) died and 5 (10%) were discharged due to clinical improvement. The patients who died showed a mean glucose of 169 mg/dl upon admission and 167.5 mg/dl upon death, while the patients who recovered had a mean central glucose of 136.5 mg/dl upon admission and 125.5 mg/dl upon discharge. These findings are consistent with the results reported by Iacobellis et al. [16]: patients with glucose >166 mg/dl have a poor prognosis compared with patients with adequate glycemic control.

The glucose levels (Figure 1) are consistent with the results of previous mortality studies in patients with hyperglycemia [16,46], especially in respiratory viral diseases, where the greatest risk of death in these patients is glucose >180 mg/dl [16,19,20,21], and which we can relate to the oxygen saturation levels. The patients who died due to COVID-19 complications were admitted with a mean oxygen saturation of 81%, which declined markedly to 68% upon death. The recovered patients were also admitted with severe hypoxemia (mean SpO_2_ of 88%), but this condition improved upon discharge (mean SpO_2_ of 92%). The difference in oxygen saturation between the groups could indicate that sustained hyperglycemia exerts a direct adverse effect on the lung. Various mechanisms may affect lung elasticity and the exchange of oxygen and carbon monoxide, which could be reflected in the patients who died due to COVID-19 complications: 100% of those patients required supplemental oxygen therapy, of which 78.6% was by means of mechanical ventilation. Among the recovered patients, who maintained glucose levels <180 mg/dl, 81.8% required supplemental oxygen therapy, of which only 9% was via mechanical ventilation.

Regarding oxygen saturation in patients with DM, there is no direct information available, but we can perform an indirect analysis by using lung function and complications related to DM, as in the study by Chelsey George et al. [47]. Those authors analyzed the risk of emphysema, chronic obstructive pulmonary disease (COPD), chronic bronchitis and asthma in patients with type 1 diabetes mellitus (T1DM), type 2 diabetes mellitus (T2DM) and patients without DM. Patients with DM had a higher risk of experiencing any of the above-mentioned respiratory conditions than patients without DM, probably due to a decrease in lung elasticity and carbon monoxide transfer capacity, which results in altered oxygen saturation [47]. In our analysis, we found that patients who died were admitted with severe hypoxemia, with a mean SpO_2_ of 83.5% that worsened to 65% upon discharge, while recovered patients, with stable glucose levels and with a tendency to improve upon discharge, showed a mean SpO_2_ upon admission of 91.5% and 93% upon discharge, which supports the importance of adequate glycemic control in patients with COVID-19.

Over-aggregated respiratory infections are also a latent problem in patients with hyperglycemia. Delamaire et al. [48] reported that patients who had sustained hyperglycemia presented greater alteration in the function of polymorphonuclear cells and opsonization, increasing the risk of over-aggregated in-hospital pneumonia by 60% [48]. In our study, this risk might also have been present and it might have been related to the presence of over-aggregated pneumonia diagnosed in 21.4% of the deceased patients and only 4.5% of the recovered patients.

There were significant differences in our in vitro experiments to assess the effect of hyperglycemia on cell viability and *ACE2* gene expression. Specifically, there was a decrease in the viability of cells treated with 200, 400, 800 or 1000 mg/dl glucose compared with control cells (*p* < 0.05). Regarding the *ACE2* expression level measured by qRT-PCR, treatment with 200 mg/dl for 72 h reduced the *ACE2* expression level, but higher concentrations markedly increased *ACE2*, with a peak at 800 mg/dl glucose. These data suggest that hyperglycemia regulates the expression and/or function of *ACE2* via some regulatory mechanism that modifies the expression of the different molecular pathways as a compensatory mechanism for the damage generated by hyperglycemia [21,28,49]. The previous results and the hypothesis of Chelsey et al. [47] may be related to the results obtained in our cell viability assays, where exposure to high glucose for >72 h affects the viability of pneumocytes in vitro. This phenomenon could imply an alteration in the pulmonary parenchyma and the exchange of carbon monoxide via diverse mechanisms influenced by hyperglycemia [47].

Glycation of *ACE2* can play a very important role in patients with hyperglycemia and COVID-19. Considering that *ACE2* is a metalloprotease with a long half-life, it can be glycated easily by enzymatic and no enzymatic mechanisms in patients with sustained or chronic hyperglycemia, thus favoring the formation of advanced glycation end products (AGEs), mainly pentosan and carboxymethyl-lysine (CML) [28,50]. Those compounds have been shown to participate in pulmonary diseases and exert various effects, including a decrease in thrombomodulin that may be related to disseminated vascular coagulation, proposed as one of the mechanisms involved in COVID-19, and D-dimer elevation [50,51]. AGEs can also generate aberrant protein cross-linking due to the glycation of type IV collagen fibers, which causes greater vascular permeability and could lead to pulmonary edema. Moreover, glycation of type I collagen fibers could trap soluble molecules and IgG in the extracellular matrix. This phenomenon may decrease the proliferation of leukocytes and produce greater fibrosis of the lung parenchyma, decreasing its elasticity, alongside the decrease in the susceptibility of these proteins to be proteolyzed [50].

The combination of these pathophysiological alterations would lead to acute respiratory distress syndrome with increased inflammation due to an uncontrolled increase in cytokines and pro-inflammatory factors, generating greater pulmonary edema and hypoxia with a worse clinical picture due to SARS-CoV-2. The state, alongside the multiple organ failure also present in these patients, significantly increases the risk of death.

## 5. Conclusions

There is a strong relationship between sustained hyperglycemia, complications and death from COVID-19. Patients who died had serum glucose >160 mg/dl, greater need for mechanical ventilation and low oxygen saturation, which increased the risk of death. There was a marked negative effect of sustained hyperglycemia on the viability of cultured pneumocytes exposed to ≥200 mg/dl glucose, and these findings may correspond to the clinical findings of lower oxygen saturation and, therefore, greater requirement for supplemental oxygen and mechanical ventilation in deceased patients. In addition, the change in the *ACE2* expression level based on the glucose concentration and exposure time could indicate some counter-regulatory mechanisms that affect the expression or function of *ACE2*. These mechanisms are still unknown and must be investigated to gain a better understanding of this complex system, with the aim of being able to offer patients better preventive and therapeutic interventions.

## Figures and Tables

**Table 1:** General characteristics of hospitalized patients with COVID-19 included in the study.

Variable	COVID-19 Outcome (*n* = 50)		*p*-Value	OR (95% CI)
	Death (*n* = 28)	Recovery (*n* = 22)		
Sex *n* (%)				
Male	9 (42.9)	12 (57.1)	0.616	-
Female	19 (65.5)	10 (34.5)		
Age (years)	59.4 ± 14.1	55.9 ± 13.9	<0.001 *	-
Hospital Stay (days)	21 ± 18	10 ± 8	0.004 *	
Symptoms *n* (%)				
Fever	9 (32.1)	10 (45.5)	0.503	0.6 (0.2–1.8)
Cough	8 (28.6)	12 (54.5)	0.116	0.3 (0.1–1.1)
Cefalea	6 (21.4)	9 (40.9)	0.238	0.4 (1.1–1.4)
Dyspnea	13 (46.4)	14 (63.6)	0.354	0.5 (0.2–1.6)
Tachypnea	13 (46.4)	12 (54.5)	0.776	0.7 (0.2–2.2)
Tachycardia	3 (10.7)	2 (9.1)	1	1.2 (0.2–7.9)
Chest pain	2 (7.1)	2 (9.1)	1	0.7 (0.1–5.9)
Diarrhea	4 (14.3)	1 (4.5)	0.368	3.5 (0.4–33.8)
Asthenia	6 (21.4)	5 (22.7)	1	0.9 (0.2–3.6)
Adynamia	6 (21.4)	5 (22.7)	1	0.9 (0.2–3.6)
Myalgia	3 (10.7)	7 (31.8)	0.084	0.3 (0.06–1.1)
Arthralgia	2 (7.1)	7 (31.8)	0.032 *	0.2 (0.03–0.9)
Anosmia	0 (0)	2 (9.1)	0.189	----
Dysgeusia	0 (0)	4 (18.2)	0.032 *	----
Odynophagia	2 (7.1)	1 (4.5)	1	1.6 (0.1–19.1)
General discomfort	3 (10.7)	2 (9.1)	1	1.2 (0.2–7.9)
Rhinorrhea	4 (14.3)	2 (9.1)	0.638	1.7 (0.3–10.1)

* *p* < 0.05.

**Table 2:** Comorbidities and clinical variables of hospitalized patients with COVID-19 included in the study.

Variable *n* (%)	COVID-19 Outcome (*n* = 50)		*p*-Value	OR (95% CI)
	Death (*n* = 28)	Recovery (*n* = 22)		
Arterial hypertension	18 (64.3)	6 (27.3)	0.021 *	4.8 (1.4–16.2)
Obesity	6 (21.4)	2 (9.1)	0.439	2.3 (0.5–15.1)
Diabetes mellitus	10 (35.7)	5 (22.7)	0.494	1.9 (0.5–6.7)
Stress hyperglycemia	17 (60.7)	15 (68.2)	0.803	0.7 (0.2–2.3)
Supplemental oxygen	28 (100)	18 (81.8)	0.032 *	----
Over-aggregated pneumonia	6 (21.4)	1 (4.5)	0.117	5.7 (0.6–51.7)
Mechanical ventilation	22 (78.6)	2 (9.1)	<0.001 *	36.7 (6.6–202.9)
HG > 160 mg/dl UA	14 (50)	6 (27.3)	0.181	2.7 (0.8–8.8)
HG > 160 mg/dl UD	17 (60.7)	5 (22.7)	0.016 *	5.3 (1.5–18.4)

HG, hyperglycemia; UA, upon admission; UD, upon discharge. * *p* < 0.05.

**Table 3:** *ACE2* mRNA expression.

Glucose Concentration	Time (hours)			*p*-Value
	24	48	72	
Control	−1.568 ± 1.872	−1.568 ± 1.872	−1.568 ± 1.872	-----
50 mg/dl	2.175 ±0.00175	−0.0322 ± 0.838	1.734 ± 0.614	0.067
100 mg/dl	0.891 ± 0.825	0.467 ± 0.378	−0.43 ± 0.204	0.138
200 mg/dl	0.234 ± 0.0589	1.193 ± 0.644	−1.473 ± 0.608	0.03 *
400 mg/dl	0.698 ± 0.704	−1.178 ± 0.204	2.375 ± 0.922	0.031 *
800 mg/dl	0.717 ± 0.00902	−0.672 ± 0.129	9.91 ± 1.312	0.001 *
1000 mg/dl	−0.658 ± 1.346	1.13 ± 2.048	3.172 ± 2.339	0.29

Data are expressed as the mean ± standard deviation of *ACE2* mRNA expression for each glucose concentration and exposure time. Expression was increased significantly in cells treated with 200, 400 or 800 mg/dl glucose, with maximum expression in cells treated with 800 mg/dl glucose for 72 h. Statistical analyses consisted of Kruskal–Wallis one-way analysis of variance on ranks, followed by multiple comparisons versus control group (Dunnett’s method); * *p* ≤ 0.05.

## Data Availability

Data that support the findings of this study are available from the corresponding author [M.L.M.-F.], upon reasonable request.

## Supplementary Materials

The following supporting information can be downloaded at: https://www.mdpi.com/article/10.3390/jpm12050805/s1, Figure S1: Standard and melt curves for *ACE2* and *RLP0*.
