# Supplementary material for: Sustained Hyperglycemia and Its Relationship with the Outcome of Hospitalized Patients with Severe COVID-19: Potential Role of ACE2 Upregulation
Source: J Pers Med. 2022 May 17;12(5):805. doi: 10.3390/jpm12050805 (PMC9147379; doi:10.3390/jpm12050805)
Supplement: Supplementary file 1 [file jpm-12-00805-s001.zip › jpm-1670875-supplementary.pdf]

Journal of Personalized Medicine

MDPI

- Supplementary material -

Sustained Hyperglycemia and Its Relationship With the Outcome of Hospitalized Patients With Severe COVID-19: Potential Role of *ACE2* Upregulation

Jose R. Vargas-Rodriguez [1], José J. Valdés Aguayo [1], Idalia Garza-Veloz [1], Jacqueline Martinez-Rendon [1], Maria del Refugio Rocha Pizaña [2], Griselda A. Cabral-Pacheco [1], Vladimir Juárez-Alcalá [1], and Margarita L Martinez-Fierro [1,*].

[1] Molecular Medicine Laboratory, Unidad Academica de Medicina Humana y C.S, Campus UAZ siglo XXI-L1, 98160, Universidad Autónoma de Zacatecas, Zacatecas, Mexico. jrvr159@gmail.com (J.R.V.-R.); josejuan104@gmail.com (J.J.V.-A.); idaliagv@uaz.edu.mx (I.G.-V.); jamare85@gmail.com (J.M-R.); gris91.edia@gmail.com (G.A.C.-P.); vladimir.j.a@uaz.edu.mx (V.J.-A.).

[2] Escuela de Ingenieria y Ciencias, Tecnologico de Monterrey Campus Puebla, 72453, Puebla, México; mrochap@tec.mx.

* Correspondence to: Margarita L Martinez-Fierro. margaritamf@uaz.edu.mx; Tel.: +52 (492) 9256690 Ext: 4535.

Journal of Personalized Medicine

Figure S1

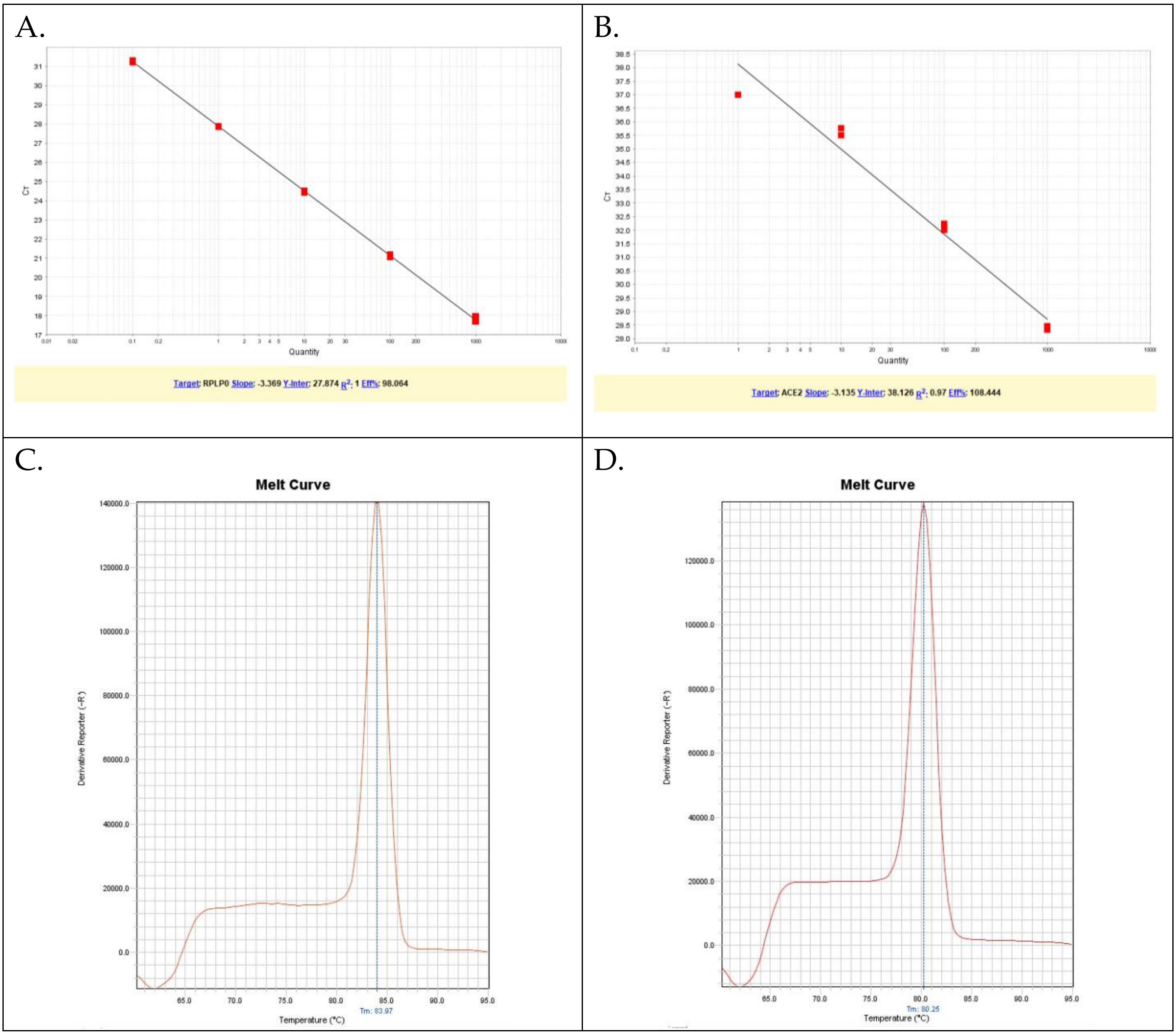

Figure S1. Standard and melt curves for *ACE2* and *RLP0*. Standard and melt curves were constructed both for *RLP0* (A-C) and *ACE2*. Standard curves were constructed using logarithmic cDNA concentrations in a range of 1000 ng to 0.01 ng. The efficiency and R^2 calculated values for *RLP0* were 98.06% and 1; whereas that for *ACE2* these values were 108.4% and 0.97, respectively. The temperatures for the melt curves were 83.97 for *RLP0* (C) and 80.25 for *ACE2* (D).
